# Physical validation of UF‐RIPSA: A rapid in‐clinic peak skin dose mapping algorithm for fluoroscopically guided interventions

**DOI:** 10.1002/acm2.12312

**Published:** 2018-03-25

**Authors:** David Borrego, Emily L. Marshall, Trung Tran, Daniel A. Siragusa, Wesley E. Bolch

**Affiliations:** ^1^ J. Crayton Pruitt Family Department of Biomedical Engineering University of Florida Gainesville FL USA; ^2^ Radiology, Division of Vascular Interventional Radiology University of Florida Jacksonville FL USA; ^3^ Present address: Radiation Epidemiology Branch Division of Cancer Epidemiology and Genetics National Cancer Institute National Institutes of Health Bethesda MD USA; ^4^ Present address: Diagnostic Physics Residency Department of Radiology University of Chicago Chicago IL USA

**Keywords:** computational phantoms, fluoroscopically guided interventions, Monte Carlo simulation, optically stimulated luminescent dosimeters (OSLDs), patient skin dose

## Abstract

**Purpose:**

The purpose of this study was to experimentally validate UF‐RIPSA, a rapid in‐clinic peak skin dose mapping algorithm developed at the University of Florida using optically stimulated luminescent dosimeters (OSLDs) and tissue‐equivalent phantoms.

**Methods:**

The OSLDs used in this study were InLight^TM^ Nanodot dosimeters by Landauer, Inc. The OSLDs were exposed to nine different beam qualities while either free‐in‐air or on the surface of a tissue equivalent phantom. The irradiation of the OSLDs was then modeled using Monte Carlo techniques to derive correction factors between free‐in‐air exposures and more complex irradiation geometries. A grid of OSLDs on the surface of a tissue equivalent phantom was irradiated with two fluoroscopic x ray fields generated by the Siemens Artis zee bi‐plane fluoroscopic unit. The location of each OSLD within the grid was noted and its dose reading compared with UF‐RIPSA results.

**Results:**

With the use of Monte Carlo correction factors, the OSLD's response under complex irradiation geometries can be predicted from its free‐in‐air response. The predicted values had a percent error of −8.7% to +3.2% with a predicted value that was on average 5% below the measured value. Agreement within 9% was observed between the values of the OSLDs and RIPSA when irradiated directly on the phantom and within 14% when the beam first traverses the tabletop and pad.

**Conclusions:**

The UF‐RIPSA only computes dose values to areas of irradiated skin determined to be directly within the x ray field since the algorithm is based upon ray tracing of the reported reference air kerma value, with subsequent corrections for air‐to‐tissue dose conversion, x ray backscatter, and table/pad attenuation. The UF‐RIPSA algorithm thus does not include the dose contribution of scatter radiation from adjacent fields. Despite this limitation, UF‐RIPSA is shown to be fairly robust when computing skin dose to patients undergoing fluoroscopically guided interventions.

## 1 INTRODUCTION

Fluoroscopically guided interventional (FGI) procedures are frequently associated with relatively high dose rates and prolonged irradiation times. There is an expressed need for physicians and clinicians to be aware of patient dose and to minimize the risk of radiation‐induced injury.^1^ Real‐time knowledge of peak skin dose has been shown to assist clinicians in managing and even reducing patient risks.^2^ In response to these issues, the authors introduced a rapid in‐clinic peak skin dose mapping algorithm developed at the University of Florida (called UF‐RIPSA) as previously presented by Johnson et al.^3^ and later modified by Borrego et al.^4^ The skin doses reported by UF‐RIPSA are evaluated based on the following expression:

(1) $$D_{\mathrm{skin}}=\left(K_{a,r}\right)\cdot \left(\beta \right)\cdot {\left(\frac{d_{\mathrm{ref}}}{d_{\mathrm{skin}}}\right)}^{2}\cdot \left(\mathrm{BSF}\right)\cdot {\left(\frac{\mu_{\mathrm{en}}}{\rho }\right)}_{\mathrm{air}}^{\mathrm{skin}}\cdot \left(AF\right)$$

where *D*_*skin*_ is the estimated dose to the skin from a single irradiation event, *K*_*a*,*r*_ is the reference air kerma reported by the radiation dose structured report (RDSR) for each irradiation event, β is a calibration factor for the KAP meter, *d*_*ref*_ is the distance to the reference point from the source location, and *d*_*skin*_ is the distance to the skin location from the source location. Other terms include *BSF*, which is a correction of backscattered x rays and secondary electrons at the skin dose point, ${\left(\frac{\mu_{\mathrm{en}}}{\rho }\right)}_{\mathrm{air}}^{\mathrm{skin}}$ is the ratio of the mass energy‐absorption coefficients (skin‐to‐air) for the relevant x ray energies considered in the dose assessment, and *AF* is an attenuation factor that accounts for the loss in energy deposition due to the presence of the tabletop and pad provided that the x ray beam intercepts these structures.

**Figure 1:**
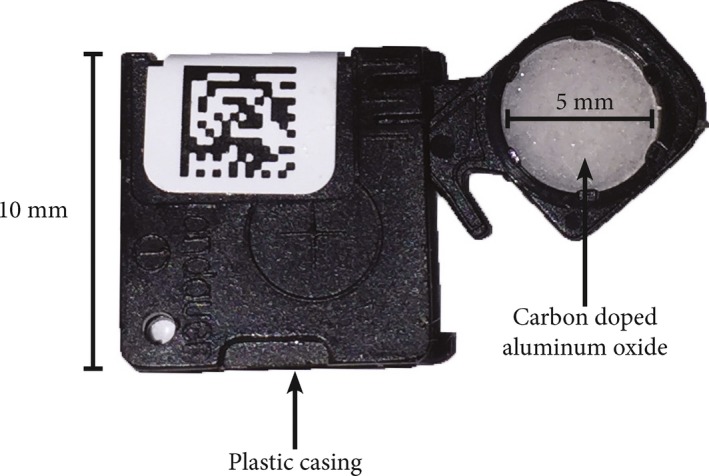
InLight^TM^ Nanodot dosimeter produced by Landauer, Inc. Plastic casing is made of tissue‐equivalent material.

Previous research groups have developed similar algorithms for skin dose mapping and reporting peak skin dose, utilizing radiochromatic film and calibrated ion chamber measurements for experimental validation. These studies report agreement with computed skin dose estimates ranging from ±4% to 6% to upwards of ±9% to 17%.^5^, ^6^, ^7^ This study presents an experimental validation of the UF‐RIPSA software by way of optically stimulated luminescent dosimeters (OSLDs). The OSLDs used in this study were InLight^TM^ Nanodot dosimeters, produced by Landauer, Inc., as shown in Fig. 1. They feature a 1 × 1 × 0.2 cm^3^ tissue‐equivalent plastic casing surrounding a disk of carbon‐doped aluminum oxide (Al_2_O_3_), which comprises the sensitive OSL material. The Al_2_O_3_ is a crystalline structure that contains valence and conduction bands. Carbon doping incorporates electron traps and hole traps within the band gap. Upon irradiation, electrons from the valence band are excited into the conduction band, generating electron/hole pairs. A fraction of these pairs recombine, but many are trapped by the energy levels within the band gap. The charge density that is trapped is proportional to the absorbed dose imparted to the OSLD. This dose record can be read by stimulating the Al_2_O_3_ with the appropriate optical wavelength of light, around 540 nm, from a light‐emitting diode (LED).^8^ Upon stimulation, trapped electrons excite toward the conduction band, and subsequently de‐excite to the valence band, producing the luminescence photons that are detected by the photomultiplier tube of an OSLD reader, such as the MicroStar^TM^ Dosimetry System (http://www.landauer.com).

Landauer, Inc., introduces a pulsed optical stimulus technique to read the luminescence in a repeating alternating sequence. This allows the reader to adjust the stimulus as weak or strong, depending on the amount of luminescence, and therefore absorbed dose, that is, detected. This distinction expands the range of absorbed dose that the reader can record. OSLD dose records can be erased when the sensitive Al_2_O_3_ material is exposed to light. Irradiation by a 150‐watt tungsten‐halogen lamp is an effective method to deplete the OSLD dose record. Lavoie et al.^9^ has previously reported on the reproducibility of results when an OSLD is reused as well as the linearity of dose–response to the x ray tube current‐time product (mAs) for diagnostic energy ranges and tissue doses.

The objective of this investigation was to validate how well UF‐RIPSA performs for fluoroscopically guided interventions. This validation was conducted with the use of multiple OSLDs to measure the dose profile on the surface of a tissue equivalent phantom irradiated by a fluoroscopic beam.

## 2 METHODS

To directly compare dose readings of the OSLDs to the results of UF‐RIPSA, these doses would have to be reported in the same domain. UF‐RIPSA used the reported reference air kerma, *K*_*a*,*r*_, and a calibration factor, β, derived from cross‐calibration of the kerma‐area product meter with values with a Radcal Model 10 × 6–6 6‐cm^3^ ion chamber. To relate the OSLDs dose readings with UF‐RIPSA, a free‐in‐air† cross‐calibration of the OSLDs with the ion chamber was computed for various x ray spectra of differing beam qualities. This study also derived Monte Carlo correction factors to adjust for any dose–response differences due to a geometry other than free‐in‐air. All measurements performed are summarized in Table 1 and discussed at length below.

**Table 1:** Summary of the experimental setup and measurement quantities

Measurement quantity	Equation, table, figure	Detector	FOV @ isocenter (cm × cm)	Geometry setup				X ray beam quality	Comments
				FIA a	Table	Pad	Phantom		
*D*_*IC*,*air*_	Equation (2)	Radcal 10 × 6–6	15 × 15, 5 × 5	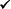				All quantities^b^	
*D*_*OSL*,*air*_	Equation (2)	Radcal 10 × 6–6	15 × 15, 5 × 5	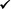				All quantities	
$D_{\mathrm{OSL}}^{\mathrm{Measured}}$	Table 2	OSLDs	15 × 15				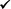	All quantities	
$D_{\mathrm{OSL}}^{\mathrm{Measured}}$	Table 3	OSLDs	15 × 15		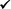		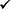	All quantities	
$D_{\mathrm{OSL}}^{\mathrm{Measured}}$	Table 4	OSLDs	15 × 15		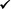	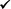	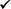	All quantities	
$D_{\mathrm{OSL}}^{\mathrm{Measured}}$	Table 5	OSLDs	5 × 5		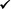		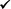	80 kVp, 0.2 mm Cu	Note^c^
$D_{\mathrm{OSL}}^{\mathrm{Measured}}$	Table 5	OSLDs	5 × 5		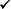	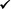	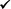	80 kVp, 0.2 mm Cu	Note^c^
$D_{\mathrm{OSL}}^{\mathrm{Measured}}$	Equation (6), Fig. 7	OSLDs	5 × 5				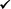	80 kVp, 0.2 mm Cu	
$D_{\mathrm{OSL}}^{\mathrm{Measured}}$	Equation (6), Fig. 8	OSLDs	5 × 5		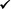	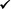	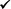	80 kVp, 0.2 mm Cu	

a Free‐in‐air.

b The peak tube potentials used were 50, 80, and 100 kVp. The amount of added filtration was varied from 0.2 to 0.6 mm Cu with no additional filtration other than inherent filtration. In total, nine beam qualities were used.

c Exposures were made with the central ray angle of incidence set at 0^0^, 30^0^, and 60^0^.

### 2.A FLUOROSCOPIC BEAM PARAMETERS

**Figure 2:**
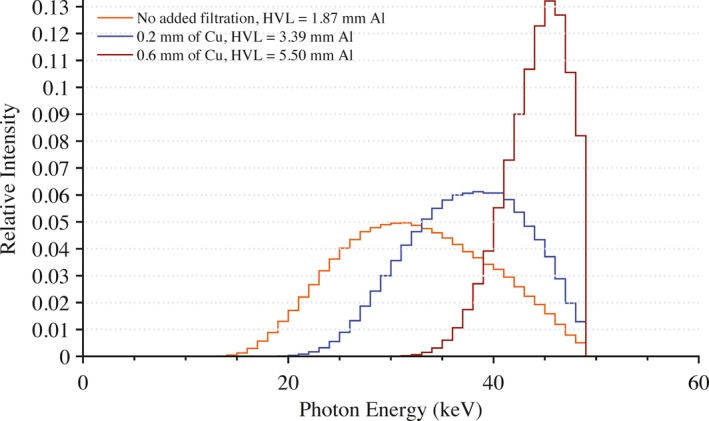
Equivalent x ray spectra used in Monte Carlo calculations to model beam qualities seen in clinic at a peak tube potential of 50 kVp with no added filtration and with 0.2 and 0.6 mm of added Cu filtration.

**Figure 3:**
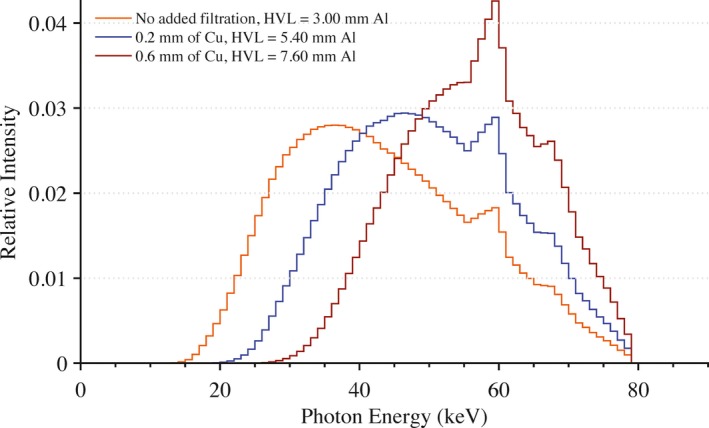
Equivalent x ray spectra used in Monte Carlo calculations to model beam qualities seen in clinic at a peak tube potential of 80 kVp for no added filtration and with 0.2 and 0.6 mm of added Cu filtration.

**Figure 4:**
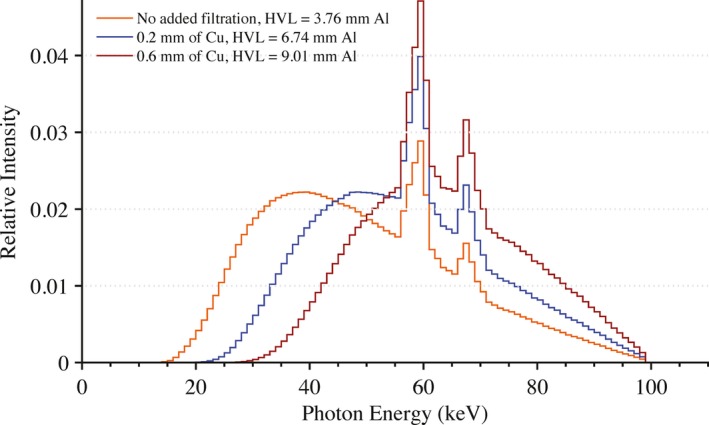
Equivalent x ray spectra used in Monte Carlo calculations to model beam qualities seen in clinic at a peak tube potential of 100 kVp with no added filtration and with 0.2 and 0.6 mm of added Cu filtration.

All exposures were performed using a Siemens Artis zee bi‐plane fluoroscopic unit. Under service mode, the pulse width was set to 500 ms with a tube current of 500 mA. This combination of pulse width and tube current produced the highest x ray yield at the most hardened beam quality investigated without triggering a safety interlock. Modifying the peak tube potential and the amount of added filtration in the beam produced various beam qualities of interest. For this study, the following peak tube potentials were used: 50, 80, and 100 kVp. The amount of added filtration in the beam was varied from between 0.2 to 0.6 mm of Cu along with no additional filtration other than inherent filtration. In total, nine different beam qualities were used in this experiment. Mathematical models of these beam qualities were developed using the HVL‐matching methodology of Turner et al.^10^ The array of equivalent x ray spectra are shown in Figs. 2, 3, 4.

### 2.B FREE‐IN‐AIR MEASUREMENTS

To characterize the OSLDs, dose measurements were taken for an ion chamber free‐in‐air and a sample of three OSLDs free‐in‐air. A Radcal Model 10 × 6–6 6‐cm^3^ ion chamber was used to assess air kerma at the isocenter of the C‐arm for each beam quality at two field sizes. The field sizes, both defined at isocenter, were 15 × 15 cm^2^ and 5 × 5 cm^2^. The same geometry was used to expose the three OSLDs. At each beam quality, cumulative doses to the ion chamber and OSLDs were recorded after five exposures. The ion chamber readings serve as energy correction factors to be applied to the OSLD readings when comparing to the results of UF‐RIPSA, which is itself calibrated to the Radcal domain — further details are given in Borrego et al.^4^

### 2.C PHANTOM MEASUREMENTS

**Figure 5:**
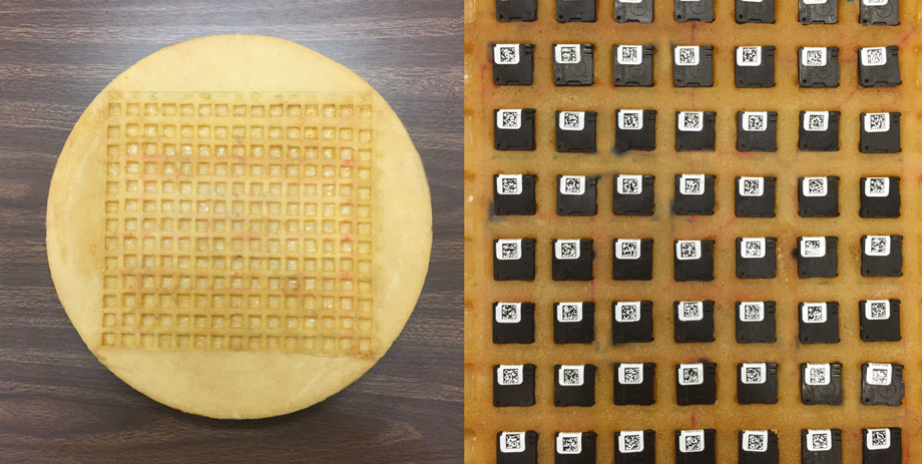
Backscattering phantom of tissue equivalent material used in this study. The grid is designed to hold the OSLDs flush with the surface of the phantom. The phantom has a diameter of 32 cm and thickness of 17.5 cm.

A cylindrical phantom of tissue‐equivalent material with a diameter of 32 cm and thickness of 17.5 cm was constructed to provide the backscattering medium for on‐phantom OSLD measurements. Details on the soft tissue equivalent substitute (STES) used in its construction can be found in Winslow et al.^11^ The phantom was designed with an embedded grid of depressions to hold the individual OSLDs, as shown in Fig. 5. The distance between the centers of the OSLDs are uniformly 1.5 cm. For this set of exposures, the beam was directed to first intersect the gridded surface of the cylindrical phantom containing the OSLDs. For each exposure, three OSLDs were placed on the plane that intersects the isocenter and is perpendicular to the beam path. These three OSLDs were then exposed five times in a field size of 15 × 15 cm^2^. This exposure was repeated using additional dosimeters for each of the nine beam qualities and then repeated for when the beam first traverses through the table, and then through both the table and pad before striking the phantom.

This irradiation geometry was repeated for a field size of 5 × 5 cm^2^ at a beam quality for a peak tube potential of 80 kVp and with 0.2 mm of added Cu filtration in the beam. At the smaller field size, the angle of incidence between the fluoroscopic beam and the OSLD plane was further varied between 0°, 30°, and 60°. These measurements were performed for when the x ray beam first traverses through the table, and then through both the table and pad before striking the phantom.

To further test UF‐RIPSA directly, new OSLDs were re‐mounted on the physical phantom. The phantom was then irradiated with a field size of 5 × 5 cm^2^ at a peak tube potential of 80 kVp and with 0.2 mm of added Cu filtration. The irradiation pattern was designed so that two distinct fields would be observed when running the corresponding UF‐RIPSA skin dose map. The OSLDs irradiated to test RIPSA were either irradiated directly on the surface of the phantom or while on the surface of the phantom with the fluoroscopic beam first traversing both the tabletop and pad. This irradiation scenario was subsequently modeled with UF‐RIPSA and the results compared to the OSLD measurements.

### 2.D ENERGY CHARACTERIZATION

In this study, Monte Carlo radiation transport simulations using the MCNPX v2.7 code^12^ were conducted for all exposure scenarios. The STES phantom was computationally modeled as ICRP Publication 89 average soft tissue.^13^ Equivalent spectra were generated with the use of the TASMIP algorithm of Boone and Seibert^14^ and the SPEKTR code of Siewerdsen et al.^15^ The MCNPX *F*6 tally for energy deposition (in units of MeV/g per source x ray photon) was used to score the energy deposition in the OSLDs. The *F*4 tally for volume flux (in units of photons/cm^2^) with a tally energy card, *En*, was used to extract the energy fluence spectra at isocenter for the different geometry setups listed in Table 1. For each MCNPX input, 10^9^ photon histories were performed on the University of Florida HiPerGator computer cluster.

### 2.E CORRECTION FACTORS

OSLD readings can be characterized partly by correction factors evaluated for each beam quality and irradiation geometry to convert dose quantities from one geometry to another. Equation (2) is a measurement based correction factor representing the ratio of the absorbed dose to the ion chamber and the absorbed dose to OSLDs, both assessed free‐in‐air. This energy‐dependent factor converts OSL free‐in‐air dose, *D*_*OSL*,*air*_, to what an ion chamber would read, *D*_*IC*,*air*_, and is thus used in the reporting of absolute skin dose for UF‐RIPSA validation.

(2) $$CF_{\mathrm{measured}}=\frac{D_{IC,air}\left(kVp,Filtration\right)}{D_{OSL,air}\left(kVp,Filtration\right)}$$

The Monte Carlo correction factor given in eq. (3) then accounts for the dose–response variation between an OSLD on‐phantom to that of an OSLD free‐in‐air. It is defined as the ratio of the Monte Carlo *F*6 tallies for energy deposition, *F*6_*OSL*_, in the OSLDS observed between the two media. Values can be given with the table and pad absent from the beam, with only the table present in the beam, or with both the table and pad present within the beam (eq. (3), respectively).

(3) $$CF_{monte\;\;carlo}=\left\{\begin{array}{c} {\left(F6_{\mathrm{OSL}}\right)}_{\mathrm{air}}^{\mathrm{phantom}}\\ {\left(F6_{\mathrm{OSL}}\right)}_{\mathrm{air}}^{phantomw/table}\\ {\left(F6_{\mathrm{OSL}}\right)}_{\mathrm{air}}^{phantom\;\;w/table\;\;and\;\;pad} \end{array}\right.$$

Equation (4) is used to predict OSLD response, $D_{\mathrm{OSLD}}^{\mathrm{predicted}}$, in a geometry other than free‐in‐air using the *D*_*OSL*,*air*_ value and *CF*_*monte carlo*_. This study explored the agreement between the predicted OSLD response and the measured OSLD response, $D_{\mathrm{OSL}}^{\mathrm{measured}}\approx D_{\mathrm{OSL}}^{\mathrm{predicted}}$.

(4) $$D_{\mathrm{OSL}}^{\mathrm{predicted}}=D_{OSL,air}\times CF_{monte\;\;carlo}$$

The general equation for skin dose reported by UF‐RIPSA, eq. (1), can be re‐arranged for OSLD measurements as shown in eq. (5). The *BSF*, μ_*en*/*ρ*_, and *AF* are accounted for in the Monte Carlo derived correction factor *CF*_*monte carlo*_, given in eq. (3).

(5) $$D_{\mathrm{phantom}}^{uf-ripsa}=K_{a,r}\times \beta \times {\left(\frac{d_{\mathrm{ref}}}{d_{\mathrm{phantom}}}\right)}^{2}\times \underset{CF_{monte\;\;carlo}}{\underbrace{\underset{}{BSF\times {\left(\frac{\mu_{\mathrm{en}}}{\rho }\right)}_{\mathrm{air}}^{\mathrm{phantom}}\times AF}}}$$

To validate UF‐RIPSA, this study explored the agreement between the measured OSLD response and corrected UF‐RIPSA values – eq. (6).

(6) $$D_{\mathrm{OSL}}^{\mathrm{measured}}\approx \frac{D_{\mathrm{phantom}}^{uf-ripsa}}{\left(CF_{\mathrm{measured}}\right)}$$

## 3 RESULTS

**Figure 6:**
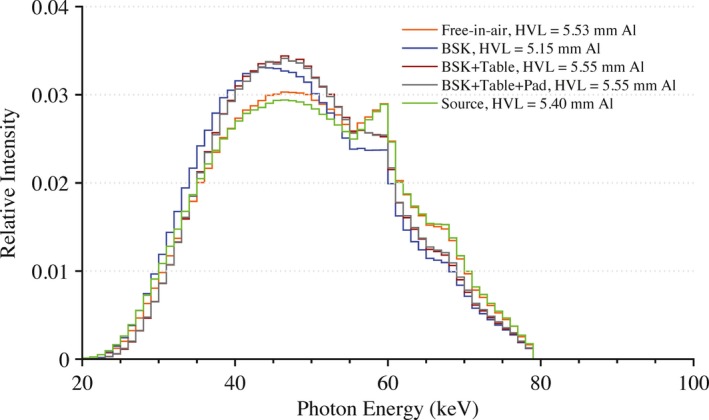
The photon energies at the OSLDs for each irradiation geometry at a peak tube potential of 80 kVp and 0.2 mm of added Cu filtration. For comparison, the initial equivalent spectrum is also shown where the BSK term in the legend indicates an energy fluence spectra with the backscattering phantom present. Similarly, the terms Table or Pad indicate that the beam first traversed these structures before the energy fluence was scored.

**Figure 7:**
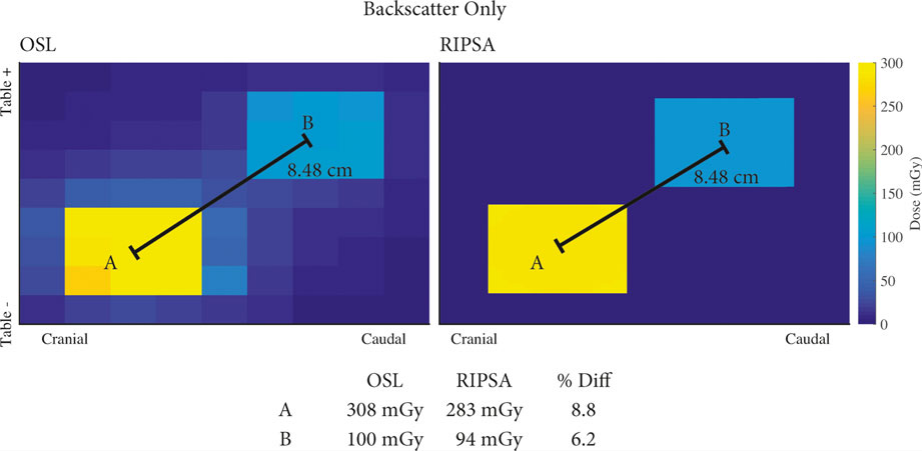
Results comparing measured values of energy imparted on the OSLDs with the best‐estimate of skin dose from RIPSA. The results from RIPSA are corrected with the use of eq. (2). Spatial orientation follows a patient recumbent, head‐first, and in a supine position.

**Figure 8:**
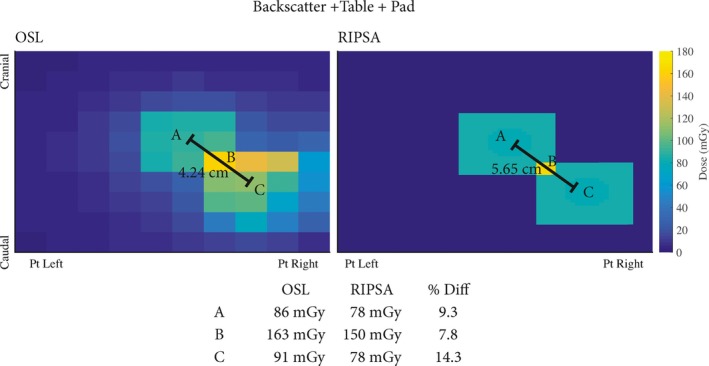
Results comparing measured values of energy imparted on the OSLDs with the best‐estimate of skin dose from RIPSA. The results from the RIPSA are corrected with the use of eq. (2). Spatial orientation follows a patient recumbent, head‐first, and in a supine position.

Applying eq. (4) for each beam quality yields predicted values of OSLD doses on‐phantom, as derived from raw OSLD measurements free‐in‐air. The OSLD doses on‐phantom along with the predicted values from eq. (4) are shown in Tables 2, 3, 4, 5. The equivalent spectra at the location of the OSLD for a peak tube potential of 80 kVp and with 0.2 mm of added Cu filtration in the beam is plotted in Fig. 6 for each geometric configuration. The results of UF‐RIPSA compared to the OSLD dose values are seen in Figs. 7 and 8. The UF‐RIPSA dose values are corrected via eq. (6) to report in the same domain as the OSLDs.

**Table 2:** Assessment of $D_{\mathrm{OSL}}^{\mathrm{Predicted}}$ on the backscatter phantom (via application of eq. (4))

Peak tube potential (kVp)	Added filtration (mm of Cu)	$D_{\mathrm{OSL}}^{\mathrm{Measured}}$ (mGy)	$D_{\mathrm{OSL}}^{\mathrm{Predicted}}$ (mGy)	Percent error (%)
50	None	65.6	61.2	−6.6
80	None	179.8	171.8	−4.5
100	None	276.5	266.1	−3.8
50	0.2	13.0	10.9	−16.1
80	0.2	61.9	57.8	−6.7
100	0.2	108.2	101.7	−6.0
50	0.6	1.7	1.6	−6.5
80	0.6	21.5	19.9	−7.5
100	0.6	43.8	45.2	3.2

**Table 3:** Assessment of $D_{\mathrm{OSL}}^{\mathrm{Predicted}}$ on the backscatter phantom with the fluoroscopic beam first traversing the table (via application of eq. (4))

Peak tube potential (kVp)	Added filtration (mm of Cu)	$D_{\mathrm{OSL}}^{\mathrm{Measured}}$ (mGy)	$D_{\mathrm{OSL}}^{\mathrm{Predicted}}$ (mGy)	Percent error (%)
50	None	57.3	54.7	−4.6
80	None	175.8	162.2	−7.7
100	None	266.8	255.8	−4.1
50	0.2	12.0	10.4	−13.4
80	0.2	62.3	57.1	−8.3
100	0.2	110.6	102.1	−7.7
50	0.6	1.6	1.6	−4.4
80	0.6	22.2	20.3	−8.7
100	0.6	45.7	46.4	1.5

**Table 4:** Assessment of $D_{\mathrm{OSL}}^{\mathrm{Predicted}}$ on the backscatter phantom with the fluoroscopic beam first traversing both the table and pad (via application of eq. (4))

Peak tube potential (kVp)	Added filtration (mm of Cu)	$D_{\mathrm{OSL}}^{\mathrm{Measured}}$ (mGy)	$D_{\mathrm{OSL}}^{\mathrm{Predicted}}$ (mGy)	Percent error (%)
50	None	49.9	47.8	−4.1
80	None	151.8	142.7	−6.0
100	None	229.3	225.5	−1.6
50	0.2	10.3	9.2	−10.5
80	0.2	57.3	50.6	−11.8
100	0.2	101.0	90.4	−10.5
50	0.6	1.5	1.4	−5.7
80	0.6	20.2	17.9	−11.2
100	0.6	41.4	41.0	−0.9

**Table 5:** Assessment of the angular dependence of $D_{\mathrm{OSL}}^{\mathrm{Predicted}}$ on the backscatter phantom with the fluoroscopic beam first traversing the table and if present, the pad (via application of eq. (4)). The table data are for beam qualities with 0.2 mm of added Cu filtration, a peak tube potential of 80 kVp, and a square field size of 5 × 5 cm^2^ at the OSLD location

Pad present	Angle of incidence (Degrees)	$D_{\mathrm{OSL}}^{\mathrm{Measured}}$ (mGy)	$D_{\mathrm{OSL}}^{\mathrm{Predicted}}$ (mGy)	Percent error (%)
No	0	45.2	48.1	6.6
No	30	43.2	46.4	7.5
No	60	36.9	36.9	−0.1
Yes	0	41.8	40.8	−2.5
Yes	30	40.7	38.5	−5.2
Yes	60	31.2	27.8	−11.0

## 4 DISCUSSION

Values of $D_{\mathrm{OSL}}^{\mathrm{Predicted}}$ (as assessed via eq. (4) with the Monte Carlo derived correction factors of eq. (3)) are compared in Tables 2, 3, 4 to values of $D_{\mathrm{OSL}}^{\mathrm{Measured}}$ for on‐phantom positioning without table/pad beam traversal, with table only beam traversal, and with both table/pad beam traversal, respectively. Percent errors are almost exclusively negative, and range in value from −16.1% to +3.2%. From the Monte Carlo simulations, the x ray spectra at the OSLD location are similar in relative intensity for all geometric configurations (see Fig. 6). The incident x ray spectrum at the OSLD locations is softest in the presence of only the backscatter phantom, and is hardest in those geometry configurations with both table/pad beam traversal. The computed HVL for the incident x ray spectra ranges from 5.15 to 5.55 mm Al equivalent. In the angular study of Table 5, percent errors range only from 6.6% to −11.0%, with increasing negative dose errors at larger angles of incidence.

The data gathered to validate the UF‐RIPSA algorithm via OSL dosimetry are shown in Figs. 7 and 8. In Fig. 7, the table and pad are absent in the irradiation geometry and the OSLDs are exposed normally to the surface of the phantom. The displacement of the fluoroscopic fields in Fig. 7 was 6 cm in both the cranial‐caudal and table height directions. The displacement between the OSLD centers is 1.5 cm in the tissue equivalent phantom of Fig. 5. Resultantly, the displacement of the two visible radiation fields from their center point values agrees between the use of RIPSA and the results of the OSLDs as expected, since the operational modular division between the field displacement and OSLDs center point displacement is zero. The recorded values by the OSLDs differ from the results of UF‐RIPSA by less than 9%, with the OSLDs always recording a higher dose value, primarily due to the inclusion of latter x ray scatter not accounted for in the UF‐RIPSA algorithm.

In Fig. 8, the beam first traverses the table and pad before normally striking the surface of the tissue‐equivalent phantom containing the OSLDs. The displacement in Fig. 8 was 4 cm in both the cranial‐caudal and left‐right directions. Due to the displacement between OSLDs centers, agreement between the center point OSLD value and the UF‐RIPSA dose points was not expected. The recorded values by the OSLDs differ from the results of UF‐RIPSA by less than 15%, with the closest agree being 7.8% in the higher dose region where the two irradiation event fields intersect spatially. UF‐RIPSA only computes a dose value to area of the patient's skin determined to be directly within the x ray beam's path, as the algorithm is based upon ray‐tracing of the RDSR‐reported reference air kerma. UF‐RIPSA will thus not compute dose contribution from photon scatter interactions within Field A onto dose point locations within Field B (Fig. 7) or Field C (Fig. 8).

## 5 CONCLUSIONS

UF‐RIPSA is a rapidly deployed algorithm for assessing skin doses incurred during fluoroscopically guided interventional procedures which is based upon a ray‐tracing of the reported reference air kerma given by the RDSR on a per irradiation event basis, with subsequent corrections for system calibration, x ray backscatter, and table and pad attenuation if required. The algorithm is thus independent of the fluoroscopic unit vendor make or model.^3^, ^4^ This study sought to validate its reporting of skin doses using custom‐built tissue equivalent phantoms and OSL dosimetry. Via Monte Carlo derived geometry‐specific correction factors, predicted values of OSLD readings were shown to agree with measured values within ~6.6% on average considering variations in both beam energy and added filtration. In the reconstruction of clinically realistic fluoroscopic projects — both spatially separate and spatially overlapping — the UF‐RIPSA algorithm was shown to agree with measured values of skin dose within a range of from −6% to −14%, with the under‐reporting of measured doses due principally to the lack of consideration of lateral photon scatter. Additional efforts are being devoted to explore ways of considering this dose contribution without the need for full‐scale Monte Carlo simulations.

## CONFLICTS OF INTEREST

There are no conflicts of interest.

## NOTE

1 Free‐in‐air in this study placed the detection instrument, Radcal ion chamber or OSLD, at isocenter in the absence of a backscattering phantom with the surface of the tabletop 25 cm below.
